# Comparative genome analysis reveals driving forces behind Monkeypox virus evolution and sheds light on the role of ATC trinucleotide motif

**DOI:** 10.1093/ve/veae043

**Published:** 2024-05-18

**Authors:** Preeti Agarwal, Nityendra Shukla, Ajay Bhatia, Sahil Mahfooz, Jitendra Narayan

**Affiliations:** Bioinformatics and Big Data, CSIR-Institute of Genomics and Integrative Biology, Mall Road, Delhi 110007, India; Academy of Scientific and Innovative Research (AcSIR), Ghaziabad, Uttar Pradesh 201002, India; Bioinformatics and Big Data, CSIR-Institute of Genomics and Integrative Biology, Mall Road, Delhi 110007, India; Bioinformatics and Big Data, CSIR-Institute of Genomics and Integrative Biology, Mall Road, Delhi 110007, India; Academy of Scientific and Innovative Research (AcSIR), Ghaziabad, Uttar Pradesh 201002, India; Department of Industrial Microbiology, Deen Dayal Upadhyaya Gorakhpur University, Civil Lines, Gorakhpur 273009, India; Bioinformatics and Big Data, CSIR-Institute of Genomics and Integrative Biology, Mall Road, Delhi 110007, India; Academy of Scientific and Innovative Research (AcSIR), Ghaziabad, Uttar Pradesh 201002, India

**Keywords:** monkeypox, microsatellite, repeats, motifs, human pathogen

## Abstract

Monkeypox (MPOX), a zoonotic disease originating in Western and Central Africa in 1970, has seen a recent surge in outbreaks across 100+ countries. A comparative analysis of 404 Monkeypox virus (MPXV) genomes revealed notable changes in microsatellite abundance and density, especially within Clades I, IIa, and IIb. Each clade exhibited unique microsatellite motifs, with twenty-six conserved loci specific to MPXV, suggesting their potential as molecular markers in diagnostics. Additionally, nine genes in the MPXV genome featured ten variable hotspot microsatellite regions associated with surface protein synthesis and host control. Notably, gene OPG153, especially at the SSR locus ‘(ATC)n’, exhibited the most pronounced variations among lineages over time and plays a role in virus pathogenesis within the host cell. These findings not only enhance our understanding of MPXV unique molecular profile but also offer valuable insights into potential pathogenic and evolutionary implications.

## Introduction

Monkeypox virus (MPXV) is a highly contagious zoonotic disease belonging to the Orthopoxvirus genus of the Poxviridae family. The virus can spread between individuals by direct contact with sores, bodily fluids, respiratory droplets, and contaminated objects. However, due to the current epidemiological situation, there is significant doubt regarding the specific dynamics of viral transmission and the extent of the outbreak (European Centre for Disease Prevention and Control 2022). The symptoms of Monkeypox (Mpox) typically manifest within a period of 6–13 days, with a maximum incubation period of 21 days following infection. The disease presents with general febrile symptoms, such as fever, headache, chills, physical weakness, and lymph node swelling. It also causes a characteristic rash (papule) on the skin and ulcers on the mucosa, accompanied by back pain and muscular aches (European Centre for Disease Prevention and Control 2022).

While monkeypox remained endemic in Africa before 2003, cases were also found in the USA in the same year (Ligon 2004). In 2017, Nigeria experienced the largest outbreak of monkeypox, with subsequent infections reported in Singapore, Israel, and the UK (Antunes, Cordeiro, and Virgolino 2022). However, the 2022 outbreak is significant as it has affected more than ninety countries, the vast majority of which are non-endemic.

The disease has gained global momentum, prompting the World Health Organization (WHO) to declare monkeypox a global emergency on 23 July 2022, following the COVID-19 pandemic (Nuzzo, Borio, and Gostin 2022). A recent study has classified MPXV into clade1/clade I, clade 2/clade IIa, and clade 3/clade Iib (Desingu, Nagarajan, and Sundaresan 2023). Clade 1 thrives solely in the Congo Basin, contributing to a significant 10 per cent mortality rate, whereas Clade 2, confined to West Africa, exhibits a lower mortality rate. Meanwhile, the extensive presence of Clade 3 has resulted in high pathogenicity across multiple countries (Americo, Earl, and Moss 2023).

Microsatellites are commonly used as genetic and molecular markers for the molecular characterization of various genomes that included bacteria (Moxon, Bayliss, and Hood 2006; Mahfooz et al. 2022a), fungi (Alotaibi et al. 2023; Kausar et al. 2024a, 2024b), and mammals (Tahoor, Khan, and Mahfooz 2019). In bacteria, these repetitive DNA sequences have been linked to pathogenicity (Mahfooz et al. 2019, 2022a) and adaptations to extreme temperatures (Mahfooz et al. 2022b). The existence of these repeats in viral genomes is also reported (Jain and Sharma 2021; Nasrin, Hoque, and Ali 2023). The above investigations prompted us to study its significance in shaping the evolution and pathogenicity of MPXV, an aspect that remains obscure. Consequently, our study aims to explore the occurrence and distribution of microsatellite in the genome sequences of various lineages of MPXV to shed light on the origin and evolution of microsatellites and their impact on MPXV adaptation within its host. In pursuit of this objective, we evaluated microsatellite occurrence, size, density, and distribution across ancient to contemporary lineages. Additionally, the study also identifies conserved and mutational hotspot sites to look into the evolutionary advantages of microsatellites variability. Moreover, microsatellite studies were used to discover unique motifs for MPXV which might be employed as diagnostic markers.

## Materials and methods

### MPXV genome sequences acquisition

**Figure 1.**
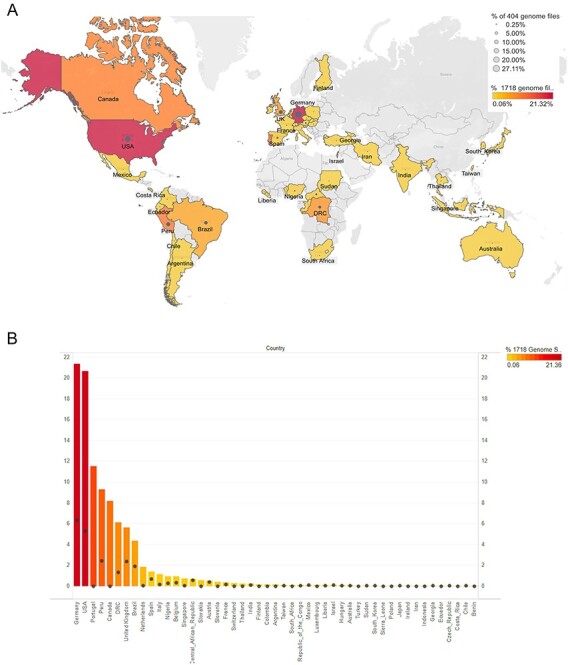
The worldwide distribution of MPXV sequencing data utilized in the present study is visualized through a map, highlighting the geographical locations of 1,718 genomes, color-coded from highest (red) to lowest (yellow) intensity. The final dataset comprising 404 genomes is represented by dots, where larger dots indicate a higher volume of sequencing data, while smaller dots depict lower sequencing data. Additionally, the graph illustrates the percentage of sequencing data obtained from various countries for both the 1,718 genomes (left Y-axis) and the subset of 404 genomes (right Y-axis).

The GISAID portal (https://www.epicov.org/epi3/frontend#486779) was used to acquire MPXV genomes in FASTA format and patient status metadata (Shu and McCauley 2017). As of 6 September 2022, we retrieved a comprehensive dataset of 1,718 genome sequences sourced from fifty-three different countries (Fig. 1) through the GISAID/EpiPox database (Elbe and Buckland-Merrett 2017; Khare et al. 2021). However, only 404 genomes with high-quality genome completeness were selected for further analysis, which are listed in Supplementary File S1.

### Genome data quality assessment

#### Genome quality check

To assess the quality of genome assembly for each of the 1,718 MPXV genomes, we utilized the CheckV v1.0.1 software and the CheckV database ‘checkv-db v1.2’. CheckV compares input genome sequences to reference genomes using its diamond database and provides an estimate of the completeness of input genomes on a scale of 0–100 per cent. By employing this methodology, we were capable of eliminating sequences that failed to satisfy the criterion for completeness, thus guaranteeing the reliability of our subsequent analysis (Nayfach et al. 2021). In order to focus on the core domain of the MPXV genome, we chose a threshold of 1.7 MB; thus, 1,623 files were chosen (Supplementary File S2). To ensure data quality, a total of 404 genome files without any illegal character and ‘N’ were selected for further processing.

### Microsatellite estimation and analysis

#### Microsatellite estimation

SSR Locator v1 was used to extract microsatellite from filtered 404 genome files (da Maia et al. 2008). This tool was configured to limit the number of repeats, with monomers set to 12, dimers to 4, trimers to 3, tetramers to 3, pentamers to 3, and hexamers to 2.

#### Microsatellite locus conservation in MPXV genomes and correlation to lineages

To evaluate microsatellite conservation, microsatellite was examined for identical motif, length, class, and repeat number at a specific locus using an in-house script (SSRmlcr.py). Flanking regions of microsatellite are known to be conserved because variations in microsatellite occur as a result of insertion and deletion of repeats (Mahoney and Magel 1996; Hosseinzadeh-Colagar et al. 2016). Therefore, we checked for the specificity of flanking regions with microsatellite in the MPXV genome using the in-house script (flanking_microsatellite_scripts.pl). Additionally, we used online NCBI BLAST v2.12.0+ to perform an extensive search in the National Centre for Biotechnology Information (NCBI) database with a customized E-value of 0.00005. Alongside conserved microsatellite, we conducted a comprehensive examination of shared and unique microsatellites across MPXV lineages using in-house scripts (upset_plot.R and SSRs.pl). To visualize shared and unique microsatellites across lineages, we employed the Upset plot, specifically using the ggupset version 0.3.0 package in R v4.2.1.

### Exploring microsatellite overlaps for functional insights and virulence prediction

To begin, we identify homologous genes (h-genes) inside all 404 MPXV genomes by aligning all 404 gene sequences with the reference gene sequences using the BLASTN v2.12.0+ program. The gene coordinates were extracted from the output file and designated as h-genes, followed by the creation of a Browser Extensible Data (BED) file based on start and stop coordinates of genes. Another BED file was created, consisting of microsatellite coordinates. Both the BED files were overlapped using BEDTools v2.30.0 to discover the microsatellite overlaps. The gene functions were determined by mapping the fragments with the reference annotation file (Supplementary File S3). The assessment of virulence and pathogenesis was carried out using the MP3 tool (Gupta et al. 2014).

### Statistical studies

We performed all statistical analysis using R (version 3.5.3) and created graphs using the ggplot2 package (Wickham 2016) in Rstudio (http://www.rstudio.com/). To compare the various classes of microsatellite among lineages, the Kruskal-Wallis test (Kruskal and Wallis 1952) was used to assess significant differences in the distributions of microsatellite classes across different lineages. Pearson correlation coefficient was applied to determine the association between genome size and number of microsatellites.

## Results

### Notable variation was observed in the relative abundance and relative density of microsatellites across different geographical regions

**Figure 2.**
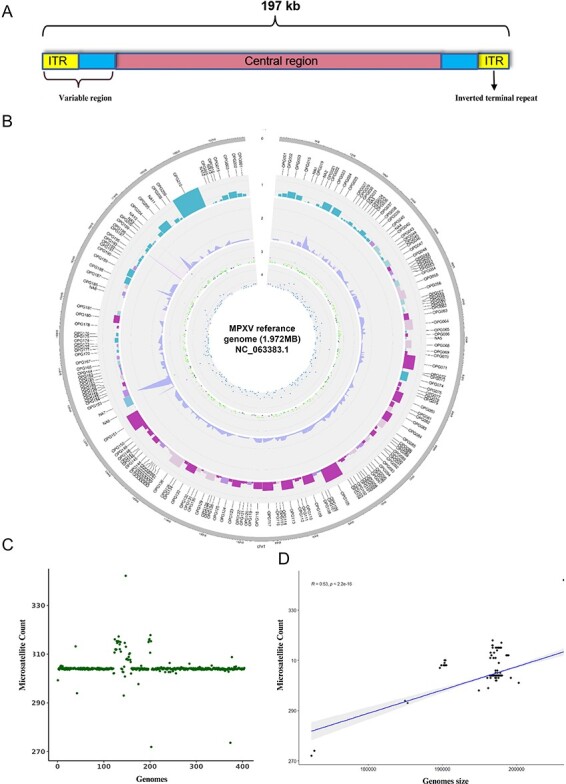
(A) Reference genome of monkeypox. The genome is a substantial 197 Kbp in length, featuring two distinct variable regions flanking the central region. These variable regions are characterized by their unique sequences and exhibit a fascinating inverted terminal repeat structure at their respective ends. (B) Microsatellite mining in reference genome: Track 0 provides an overview of the reference genome, which spans 197 Kbp in length. Track 1 showcases 190 genes, displaying their respective sizes and precise locations along the genome. The functional categories and their corresponding colors are as follows: blue green for genes involved in host modulation, light steel blue color for genes not yet annotated, violet for surface proteins, and dark pink for genes associated with replication/transcription. Additionally, light pink indicates genes that play a role in assembly and budding processes. In Track 2, the length of microsatellites at specific loci is presented through a visually informative line graph. The coding regions are represented in slate blue, while non-coding regions are displayed in light pink. Track 3 enhances the visualization by presenting the microsatellite length, with color variations indicating different classes of microsatellites. Finally, Track 4 differentiates microsatellite numbers located in coding regions (shown in blue) from those in non-coding regions (displayed in pink). (C) Average microsatellite count in the 404 dataset. On an average, 300 microsatellites were found to be present in each genome. (D) Pearson correlation graph shows positive correlation between genome size and number of microsatellite (*P*-value <2.2e^–16^).

In order to gain insights into the dynamics and distribution of microsatellites across all lineages of the MPXV genome, we used the 197 Kbp long MPXV reference annotated genome (NC 063383.1) from NCBI to compare against all 404 genomes (Fig. 2A). The MPXV reference genome can be traced back to its origin in Nigeria. It comprises 190 genes and belongs to lineage IIb B.1 (probable IIb B.1) (Fig. 2B). Microsatellite mining in the reference genome revealed that the coding region contains the majority of microsatellites (around 80 per cent). A comprehensive analysis revealed a total of 304 perfect microsatellites, exhibiting varying frequencies among different microsatellite classes: 3 monomers, 52 dimers, 127 trimmers, 8 tetramers, 1 pentamer, and 113 hexamers (Fig. 2B). Among the observed microsatellites, the highest frequencies were recorded in trimers (41.78 per cent) and hexamers (37.17 per cent), highlighting their prevalence in the reference dataset (Fig. 2B).

In our analysis, we observed that smaller genomes typically included fewer microsatellites (EPI_ISL_13158444, 172 Kbp, 272 microsatellites), whereas larger genomes exhibited higher frequency of microsatellites (EPI_ISL_13058459, 206 Kbp, 342 microsatellites). Our examination of 404 monkeypox genomes yielded 123,046 microsatellites in total, with an average of 304 ± 37 microsatellites per genome (Fig. 2C). It is evident from the data that genome size influences the frequency of microsatellites, supported by a highly significant positive correlation between genome sizes and frequency of microsatellites (*R*^2^ = 0.53 and *P*-value = 2.2e^–16^) (Fig. 2D). We utilized a simple method to compare all genomes at the microsatellite level to address genome size variation. Microsatellites’ relative density (RD) and relative abundance (RA) were calculated using microsatellites’ frequency and length per megabase (Mb). Sudan (EPI_ISL_13058459) had the highest RA (1,657.20/Mb) and Liberia (1,503.07/Mb) the lowest, with an average of 1,547.16. Similarly, we examined microsatellite density and discovered surprising ranges between 16,014.50/Mb in Liberia (EPI_ISL_13058405) to 18,597.48/Mb in Sudan. This suggests that RD varies by region, with Liberia having the lowest value and Sudan the highest in the genomes analyzed.

### Distinct variations in RA and RD of microsatellites were observed among lineages

**Figure 3.**
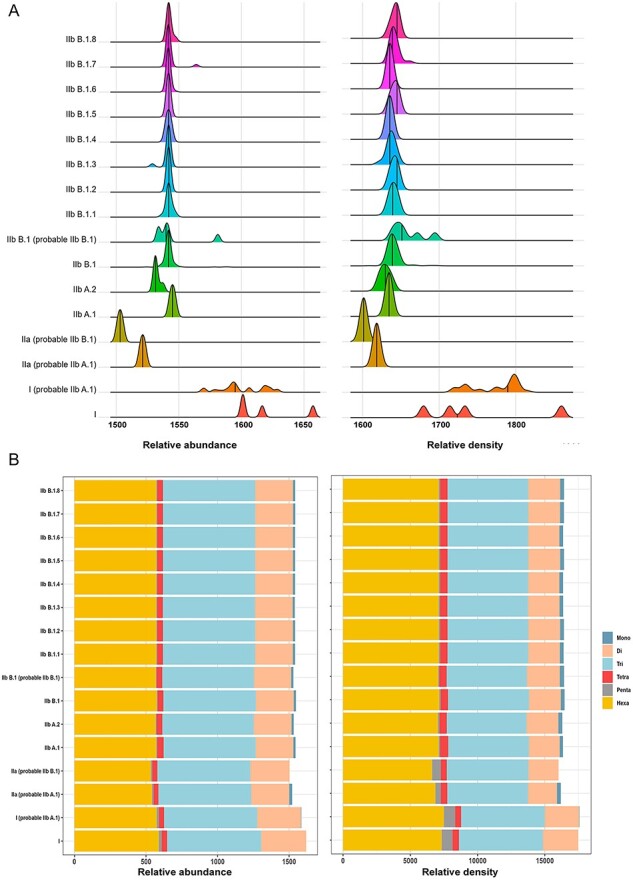
Lineage wise distribution of microsatellites. (A) RA and RD of microsatellites across sixteen lineages shown through ridgeline plot. A distinct contrast is easily discernible between ancestral and contemporary lineages. (B) Stacked plot showing the distribution of RA and RD among the different classes of microsatellites in all sixteen lineages. Within ancient lineages, pentamers were abundant, while monomers were found almost absent. Conversely, in recent lineages, the pentamer amount was reduced, and there was a notable increase in the number of monomers.

We further looked into the distribution of microsatellites in the sixteen lineages of MPOX. Lineage IIa (probable IIb B.1) displayed the lowest RA (1,503.076/Mb) and RD (16,014.5), indicating a lower concentration of microsatellites. On the other hand, lineage I (probable IIb A.1) exhibited the highest RA (1,619.069/Mb) and RD (17,729.93/Mb), signifying a higher abundance of microsatellites. These findings emphasize distinct variations in relative abundance among lineages, with lineage IIa (probable IIb B.1) exhibiting the lowest abundance and lineage I (probable IIb A.1) displaying the highest abundance among the genomes examined. The graph visually illustrates a significant difference from ancient lineages to present lineages (Fig. 3A).

**Figure 4.**
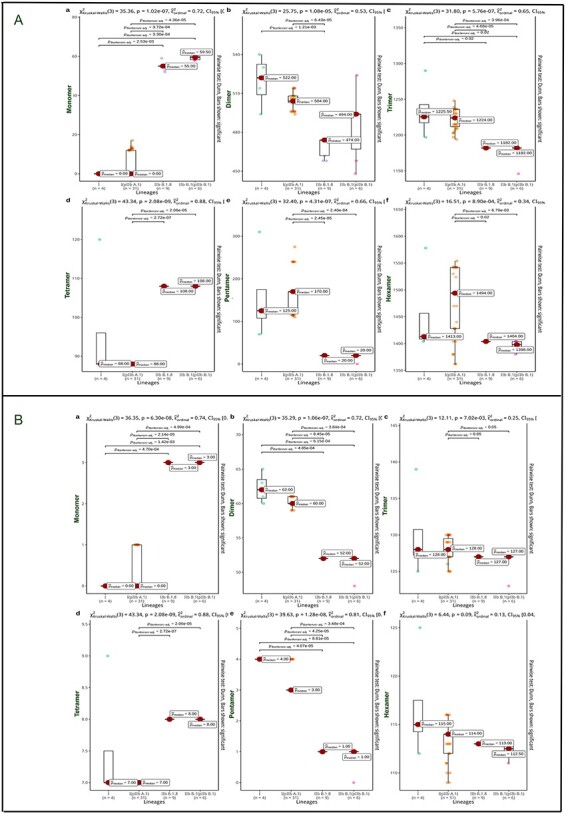
Microsatellites lineage class comparison. The Kruskal-Wallis test is used to rigorously assess the differences among multiple groups of MPXV lineages. The observed discrepancies in microsatellite classes between the ancient lineages (Lineage I, I (likely IIb A.1), the reference lineage (IIb B.1), and the recently evolved lineage (IIb B.1.8) were exactly identified by (A) microsatellite length: Lineage I (likely IIb A.1) exhibited considerable variation in monomer, dimer, trimer, tetramer, pentamer, and hexamer (B) microsatellite frequency: Lineage I showed significant variation in monomer, dimer, and pentamer. Monomer, dimer, trimer, tetramer, and pentamer variation was substantial in Lineage I (probable IIb A.1).

We performed a time-dependent comparison of microsatellite classes across all genomes. The examination of all MPXV microsatellite classes from 1962 to 2022 shows minor lineage changes (Supplementary File S4). Trimers are more common, followed by hexamers and dimers in decreasing order across all lineages. Monomers, tetramers, and pentamers are less abundant (Fig. 3B). To further investigate the classes of microsatellites, we conducted a comparative analysis between the most recent lineages (IIb B.I.6 & IIb B.1.8) and the ancient lineages (I and I (probable IIb A.1)). Two criteria, namely the length and number of microsatellites, were compared in this study by utilizing the available metadata from 404 genomes. By comparing the ancient lineages (I and I (probable IIb A.1)) with the contemporary lineages (IIb B.1.6/IIb B.1.8), we observed significant differences among the group means for the mono, di, tetra, and pentamer classes of microsatellites in the ancient and contemporary lineages by using Kruskal-Wallis test (Fig. 4A, B). The results of the post-hoc analysis using the Bonferroni correction further provided additional evidence supporting the observed significant differences among the microsatellite classes. These findings indicate that both the length and the number of microsatellites have undergone notable alterations during the course of evolution, as indicated by the significant differences observed. Moreover, the analysis of MPXV data indicates a gradual reduction in the size of pentanucleotide repeats within ancient lineages. This reduction is accompanied by a compensatory increase in the prevalence of mononucleotide repeats within the contemporary lineages over time.

### Microsatellite locus conservation in MPXV and its role as possible diagnostic markers

**Figure 5.**
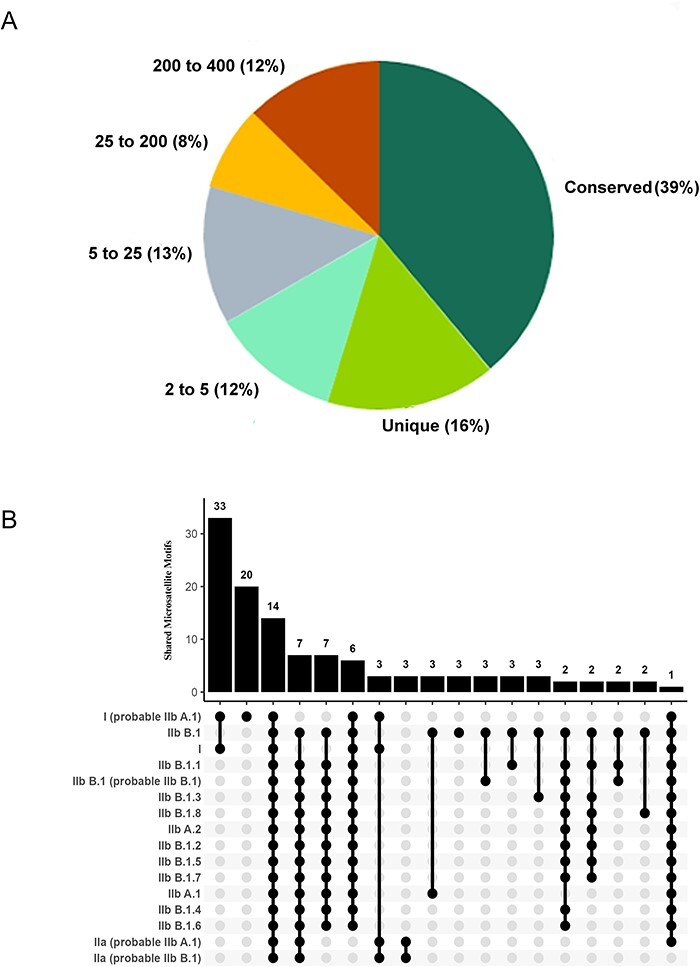
(A) Conservation of microsatellites in MPXV. Among the 318 distinct types of microsatellites discovered across 404 genomes, 39 per cent were conserved, 16 per cent were unique, and the remaining 45 per cent microsatellites were shared across MPOX genomes. (B) Shared microsatellites in various lineages: upset plot depicts that approximately 45 per cent of microsatellite types were identified as shared among diverse lineages. Notably, the highest (33) occurrence of shared microsatellites was observed between lineage I and I (probable IIb A.1). Additionally, fourteen microsatellites were found to be present across all lineages.

We explored the conservation of microsatellite locus in MPXV genomes to assess their evolutionary relationships. Among 404 genomes, we identified 318 microsatellite motifs based on criteria mentioned in methodology section IIIb. Notably, 39 per cent of these microsatellites were conserved across all genomes, while 16 per cent were unique to a single genome. Furthermore, 45 per cent of the genomes shared, with 12 per cent overlapping in 2–5 genomes and 13 per cent in 5–25 genomes. Notably, microsatellites increased their prevalence dramatically, with 8 per cent detected in 25–200 genomes and 12 per cent throughout 200–400 genomes (Fig. 5A). To assess the potential of microsatellites as virus-specific probes or primers, we expanded our analysis by including flanking sequences of up to 10 nucleotides both upstream and downstream, creating sequences of 20–30 nucleotides in total. Out of the 39 per cent conserved microsatellites (124 microsatellites), 64 microsatellites with flanks remained conserved in all genomes. Further confirming their specificity, a BLAST search revealed that twenty-six microsatellites with flanks were unique to MPXV, solidifying their exclusive presence in this organism (Table 1). This information holds immense value, as these distinctive DNA sequences can serve as crucial molecular markers or diagnostic tools for accurate identification of MPXV and hold potential for vaccine development as well (Curtidor et al. 2017).

**Table 1.** Unique MPXV regions encompassing SSRs and tailored flanking sequences: twenty-six conserved regions specific to MPXV are mentioned with their positions in the reference genome. Gene and SSRs associated with these regions are listed.

S.No.	Start position	Stop position	Conserved region	Length	Gene	SSR
1	12025	12056	CAATTTTACCAAATCTAAATCTACATCTTTAT	32	OPG023	(AAATCT)2
2	17846	17877	AATAAAAATCCATTTTCATTTTTAGCACAATA	32	Near to gene—OPG031	(CATTTT)2
3	17877	17908	ACTATTCATAATTGATATTGATGTAATATTTT	32	Gene-OPG031	(ATTGAT)2
4	20480	20511	CTTATTCATATTTTAGTTTTAGGATCGAGAAT	32	No	(TTTTAG)2
5	23044	23075	TCGGCGACAAAGTACCAGTACCGGTAATCTTG	32	OPG038	(AGTACC)2
6	24267	24298	TTCATTAAGAAGATTCAGATTCCACTGTACCC	32	No	(AGATTC)2
7	32224	32255	ATCTTTTGTATATCGCTATCGCCGCAATCACT	32	OPG049	(TATCGC)2
8	33596	33627	TACATCTTCCTTCTTATTCTTAGCGTCACAGA	32	OPG051	(TTCTTA)2
9	35249	35280	TATTTCCTCTTTCGTATTCGTAATTAGTTCTC	32	OPG054	(TTCGTA)2
10	53737	53768	TCGTTCGTAATCCATTTCCATTAGCGACTGTA	32	OPG071	(TCCATT)2
11	63550	63581	TCCTGCGTTATCCGTTTCCGTTATATACAGGG	32	OPG083	(TCCGTT)2
12	68556	68587	AACGTTCGTATATCGTTATCGTCGTATAAATT	32	Near OPG086	(TATCGT)2
13	92172	92203	CTTATTCAAGCAAAAACAAAAAACTTTACGAT	32	OPG113	(CAAAAA)2
14	126765	126796	ACATACATTTGTAAAAGTAAAAAAGAAAAACA	32	OPG148	(GTAAAA)2
15	132821	132852	TCTTTTGTAGAACTTTAACTTTTTCTTTCTCA	32	No	(AACTTT)2
16	164969	165000	TGGAAAACTTTAACGATAACGAACTGACCACA	32	OPG189	(TAACGA)2
17	99684	99711	TCAATTGATGTTTTTGTTTTCGTCAAAC	28	OPG118	(TGTTTT)2
18	44741	44768	TATTAAAGTATCGATATCGATCTCGTCT	28	OPG064	(TATCGA)2
19	164564	164589	CAATTACTAAACATAAACAACATAAT	26	OPG189	(TAAACA)2
20	125557	125580	CAATCATCATCGTCATCGATAACT	24	OPG146	(TCATCG)2
21	29205	29228	CTATATCATGTACATGTACATAAT	24	OPG045	(CATGTA)2
22	150645	150666	TGTATCCATTCCCATTCAAATC	22	No	(CCATTC)2
23	175965	175984	GAGAATGAATATGAATTCTA	20	OPG205	(ATGAAT)2
24	156301	156320	GGTGGTTTAAGTTTAAAAAA	20	No	(GTTTAA)2
25	82602	82621	AAAGGTAAATGTAAATTCAG	20	OPG105	(GTAAAT)2
26	52889	52908	AAGATAGTTTTAGTTTCCAA	20	OPG071	(TAGTTT)2

We also wanted to see if there was any lineage-specific conservation of microsatellites in the MPXV genomes. As depicted in Fig. 5A, 45 per cent of microsatellites were found shared among 404 genomes where lineage I and lineage I (probable IIb A.1) have thirty-three microsatellites in common, the highest across all lineages. Likewise, fourteen microsatellites were conserved across all of the MPXV lineages. A small percentage of microsatellites were discovered to be specific to lineages, indicating their uniqueness in terms of lineage-specific microsatellites (Fig. 5B). The results indicate that certain microsatellites were initially shared among ancestral lineages; however, over the period, these microsatellites underwent evolutionary changes within new lineages or were entirely lost, highlighting the dynamic nature of microsatellites over evolutionary periods.

**Figure 6.**
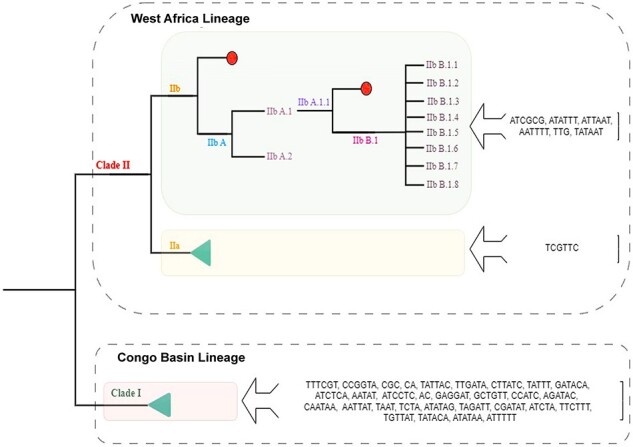
Clades-specific microsatellite motifs. Phylogenetic tree displaying MPXV lineages up to the IIb B.1.8 variant. Clades 1, 2, and 3 are represented by red, yellow, and green boxes, respectively. Unique motifs from each clade are displayed on the right. Green triangles represent lineage groups, while red circles represent dead ends.

After considering conserved microsatellite loci across lineages, we further delved deeper into unique microsatellite loci that are exclusive to particular countries and lineages. In this context, ‘unique loci’ denotes microsatellite characterized by a specific combination of motif and length, which distinguishes them from the term ‘unique motif’. We found fifty unique microsatellite loci in seventeen countries across eleven lineages. The unique count of microsatellites, ranked from highest to lowest, is as follows: Sudan (10) > DRC (8) > Liberia (6) > Central African Republic and USA (5) > Nigeria, Germany, Brazil, and Austria (2) > UK, India, Spain, USA, Republic of Congo, Hungary, Peru, Mexico, and Italy (1). Around 50 per cent of unique microsatellite loci belong to the ancient lineages, specifically I and I (probable IIb A.1). It is worth highlighting that as we move from ancient to contemporary lineages, there is a noticeable loss as well as gain of distinct microsatellite loci. We further investigated the possibility of assigning unique microsatellites to the different phylogenetic clades of MPOX. Following the microsatellite isolation criteria outlined in section IIIa, we observed that Clade I had thirty motifs, Clade 2 had one motif, whereas Clade 3 had seven motifs (Fig. 6).

### Hotspot microsatellite loci significantly influence lineage evolution and potentially contribute to enhanced virulence

**Figure 7.**
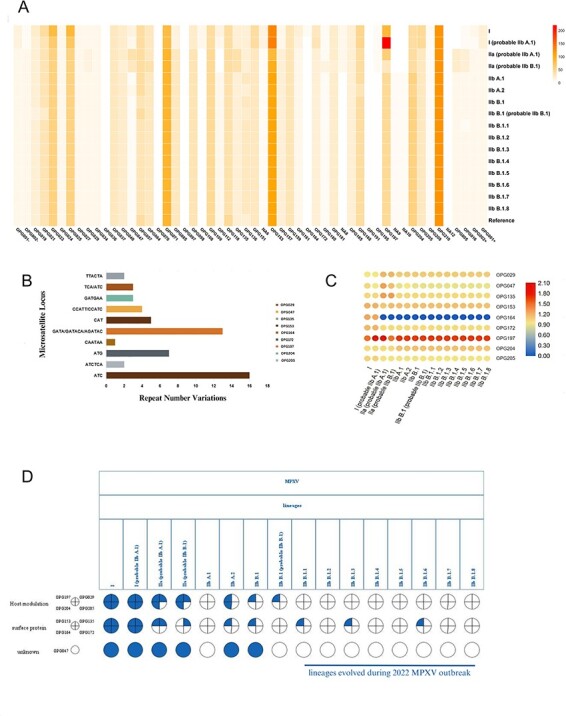
Variable hotspot microsatellite loci. (A) Heatmap displaying microsatellite length variation in fifty-four genes across sixteen lineages. (B) A total of 10 microsatellites hotspot loci were detected in nine genes. The most substantial changes in microsatellite length were recorded in genes OPG153 and OPG197, where notably gene OPG153 featured two microsatellite hotspot loci. (C) A comprehensive heatmap depicting microsatellite hotspot genes across all sixteen lineages. In addition to the reference genome, this visualization only included genomes with variants. The heatmap discernibly reveals the gradual disappearance of microsatellite in OPG164 over successive generations. (D) The plot depicts the functional role of nine hotspot genes, as well as lineages which have differences from the reference.

We conducted a comprehensive investigation of microsatellites within MPXV genes, revealing variations in fifty-four genes (Fig. 7A). Notably, thirty-four genes exhibited significant differences (≥5 genomes), with twenty-five showing point mutations and nine displaying repeat number variations. Additionally, our analysis identified ten microsatellite hotspot loci with varying repeat lengths in nine genes (OPG029, OPG047, OPG135, OPG153, OPG164, OPG172, OPG197, OPG204, OPG205) within the MPXV genomes (Fig. 7B). We observed that the number of DNA repeats varied across these motifs, with some having as few as two and others having as many as sixteen repeats. Out of nine genes, two genes in particular, gene OPG153 and gene OPG197, had highly variable microsatellite motifs with repeat numbers of 16 and 13, respectively. To gain further insights, we analyzed all the genomes exhibiting variations in microsatellite loci in nine genes across sixteen lineages (Fig. 7C). The heatmap shows the gradual disappearance of microsatellite in gene OPG164 over successive generations. An evident contrast in the lengths of microsatellites across various genes is observed between ancient and contemporary lineages. Ancient lineages are marked by longer microsatellite repeats, while shorter microsatellite repeats are prevalent in contemporary lineages. This observation underscores the enduring influence of microsatellites in intricately modulating the adaptive potential of MPXV over extended evolutionary timeframes

### The microsatellite locus ‘ATC’ appears to display a substantial impact throughout the evolutionary trajectory of MPXV

Upon investigating the functional aspects of the nine genes (see above) within the reference genome, we made intriguing observations. Four of these nine genes played an essential role in host modulation, while the other four were involved in surface protein modification, and one gene remains unidentified (Fig. 7D). Further, we determined the role of these genes in pathogenesis and interestingly, gene OPG029 was predicted to be directly involved in pathogenesis which clearly establishes the role of microsatellite in this process, while the rest of the eight genes were predicted to be likely pathogenic. Through our analysis, we have illuminated the prominence of gene OPG153, specifically within the microsatellite locus ‘ATC’, which exhibits the most substantial variations across all eight lineages. Remarkably, only this particular locus demonstrates alterations spanning both ancient and contemporary lineages (Supplementary Fig. S1). Importantly, the functional involvement of this gene in surface protein formation amplifies its significance in underscoring its potential importance in virulence (Fig. 7D).

## Discussion and conclusion

The presence of microsatellites in viral genomes can lead to rapid changes in its motif’s repeat number due to their inherent instability (Yamamoto and Imai 2015). This rapid change can facilitate genetic drift, allowing viral populations to adapt quickly to changing environments or host immune pressures. Our data revealed that among different countries, the MPXV strain originating from Sudan (clade 1) exhibited the highest RA of microsatellites, while the strain from Liberia (clade 2) displayed the lowest RA. Since Sudan is in North Africa and Liberia is in West Africa, perhaps the different geographic locations of these two strains explain this discrepancy. The amount of A + T or G + C content in the genome also plays a major effect in determining the number of microsatellites found (Tian, Strassmann, and Queller 2011). Previous research has shown that changes in microsatellite frequency are associated with changes in either AT or GC content of the genome (Mahfooz et al. 2015, 2017). Since the MPXV genome is AT rich, we might speculate that the higher RA in Sudan may be due to the higher AT composition of the genome. Among the classes, trinucleotide repeats were found to be the most abundant across all lineages, followed by hexanucleotide repeats. This event, while expected, is significant because of the possible translation of tri- and hexanucleotide repetitions into amino acids (Li et al. 2004). Furthermore, the small size of the viral genome highlights the limited ability for gene accumulation, emphasizing the predominance of these specific repeat motifs. While analyzing the different lineages, we observed that the ancient lineages have more RA and RD as compared to the recent ones. The possible reason behind the higher RA and RD in ancient lineages could be attributed to the presence of a significant number of penta nucleotide repeats within them. After a close observation of our data, we can hypothesize that by the time of evolution, these penta nucleotide repeats gradually decreased and were replaced by monomeric repeats in the upcoming lineages.

In our motif conservation study, we observed that 39 per cent of microsatellites were conserved among the MPXV genomes. The surprising observation could be explained by the strategic arrangement of these microsatellites within the core areas of MPXY, where they serve critical roles in coordinating fundamental cellular processes required for its survival. It is possible that, due to their critical functions, the evolutionary flexibility of these microsatellites has been severely limited, potentially restricting their ability to undergo large modifications over time (Metzgar, Bytof, and Wills 2000). Thus, on the other hand, the 16 per cent motifs that were found to be unique in the genome may be located in the regions that are actively involved in host adaptation or pathogenicity. There have been several studies that suggest that the evolution of monkeypox may have been influenced by human Apolipoprotein B mRNA editing enzyme catalytic polypeptide 3 (APOBEC3). APOBEC3 deaminase acts as a component of the innate immune system and limits the entry of virus elements by mutating nucleotides G to A and C to T on complementary strands of freshly synthesized viral genomes (Delamonica et al. 2023; O’Toole et al. 2023). In addition to variation resulting from APOBEC3 deaminases, here we present evidence that SSRs could play a vital role in driving MPOX evolution and adaptation. Increase or decrease in the length of SSR in particular genes due to DNA polymerase slippage and strand mispairing during replication can lead to variations in a gene, which in turn results in adaptive evolution (Mahfooz et al. 2022b). In a recent study to investigate the functional implications of SSR insertions, researchers used three-dimensional protein structure modeling on a diguanylate cyclase (DGC) gene that encodes a protein containing SSRs. Interestingly, when the SSR sequence was removed, the protein folded incorrectly and became more unstable, emphasizing the importance of SSRs in maintaining optimal protein structure and function (Mahfooz et al. 2022b).

Our data also indicate that the MPOX strain showed changes in the number of repeats in nine genes in which four genes were involved in surface protein formation whereas four genes were found to assist host modulation and the function of one of the gene was unknown. In this context, the gene OPG153 exhibits the ATC motif, highlighting significant variation across various MPXV lineages (Fig. 7B). On the sense strand, ATC codes for aspartic acid that has been reported to play an important role in viral entry to the host. Previously, it was shown that the coxsackie virus A9 (CAV9) uses a combination of arginine-glycine-aspartic acid (RGD) motifs to penetrate mice cells. It has been proven that changes to the RGD-containing areas can be used to examine the role of this tripeptide in CAV9 pathogenesis in mice. As a result, we can hypothesize that changes in aspartic acid repeats may aid in entrance into human host cells and contributing to virulence of MPXV (Harvala et al. 2003). This suggests that the ATC pattern may have played a key role in the evolutionary process of MPXV, potentially contributing greatly to its development and pathogenicity. Additionally, another gene (OPG153) of MPXV protein codes for P4c protein which is crucial for viral pathogenesis in the host cell. This protein is essential to the complicated structure of intracellular mature virus (IMV) surface tubules, which are the most infectious viral particles (Chung et al. 2006; Moss 2012). It binds virus to laminin and directs mature virus (MV) into A-type inclusion bodies (McKelvey et al. 2002; Chiu et al. 2007). This protein significantly shapes intracellular virus surface structures fundamental to viral infectivity. By disrupting host signaling mechanisms, these proteins impede immune responses, hindering interferon reactions, antigen presentation, and cytokine production. This interference fosters more efficient virus proliferation. The observed motif variations, akin to bacterial adaptive strategies, suggest viruses employ similar mechanisms to swiftly adapt in changing environments, ensuring their survival and evolution.

In recent observations derived from the most current data accessible through GISAID, a prominent pattern has become evident, signifying the frequent evolutionary changes within lineages IIb B.1 and B.1.3. Concurrently, we have detected variations within the ATC locus of OPG153 across lineages IIb B.1, IIb B.1.1, IIb B.1.3, and IIb B.1.6 (Fig. 7D). These findings suggest that the microsatellite locus ATC may have exerted a significant influence on the evolutionary dynamics of MPXV, potentially playing a substantial role in its development and adaptive processes. However, comprehensive research is imperative to thoroughly elucidate and investigate the exact contribution of these variations to virulence. While there are many PCR-based diagnostic primers available, most of them lack specificity for monkeypox and instead target other orthopoxviruses (Kulesh et al. 2004; Li et al. 2006) or rely on costly TaqMan assays (Li et al. 2010). Therefore, the markers produced from the conserved areas of 404 MPXV could be helpful tools for the low-cost precise diagnosis of MPXV.

## Supplementary Material

veae043_Supp

## Data Availability

Data, in-house scripts, and any other material are completely open to the public. All codes may be found at https://github.com/PRTGRWL/MPXV_Scripts. All local data related to MPXV genomes are available in supplementary files.
