# Supplementary material for: Comparative genome analysis reveals driving forces behind Monkeypox virus evolution and sheds light on the role of ATC trinucleotide motif
Source: Virus Evol. 2024 May 18;10(1):veae043. doi: 10.1093/ve/veae043 (PMC11141602; doi:10.1093/ve/veae043)
Supplement: veae043_Supp [file veae043_supp.zip › suppl_data/Supplementary file_4.docx]

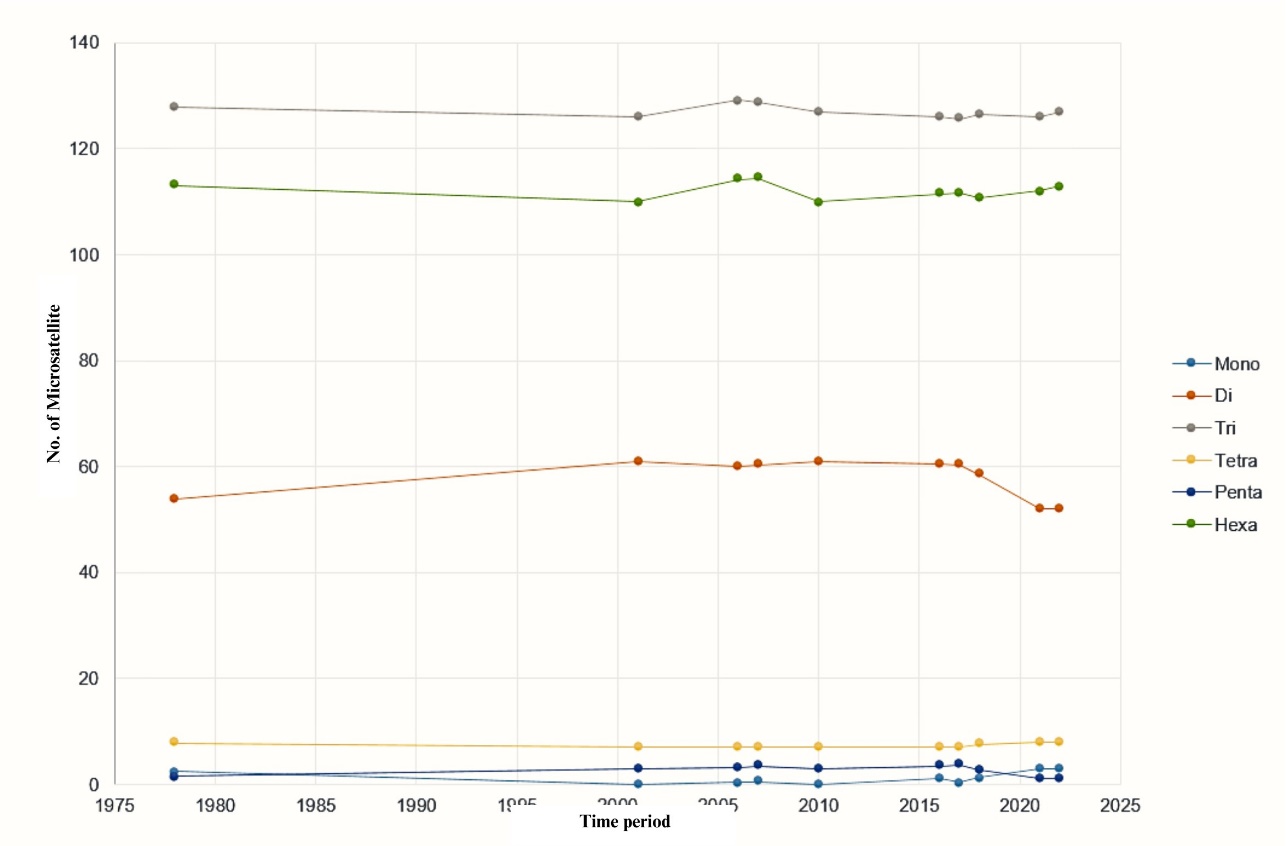


**Supplementary figure 1: Distribution of MPXV microsatellites across time period (1962- 2022).** An informative line graph vividly illustrates the temporal distribution of mono-, di-, tri-, tetra-, penta-, and hexa-nucleotide repeats spanning approximately 60 years. The graph provides a clear visual representation of how the frequency of these repeats changes over time, offering valuable insights into any trends or patterns that might emerge within this extensive timeframe.
