# Supplementary material for: Comparative genome analysis reveals driving forces behind Monkeypox virus evolution and sheds light on the role of ATC trinucleotide motif
Source: Virus Evol. 2024 May 18;10(1):veae043. doi: 10.1093/ve/veae043 (PMC11141602; doi:10.1093/ve/veae043)
Supplement: veae043_Supp [file veae043_supp.zip › suppl_data/Supplymentary_Figure_1.docx]

**Supplementary image 1**


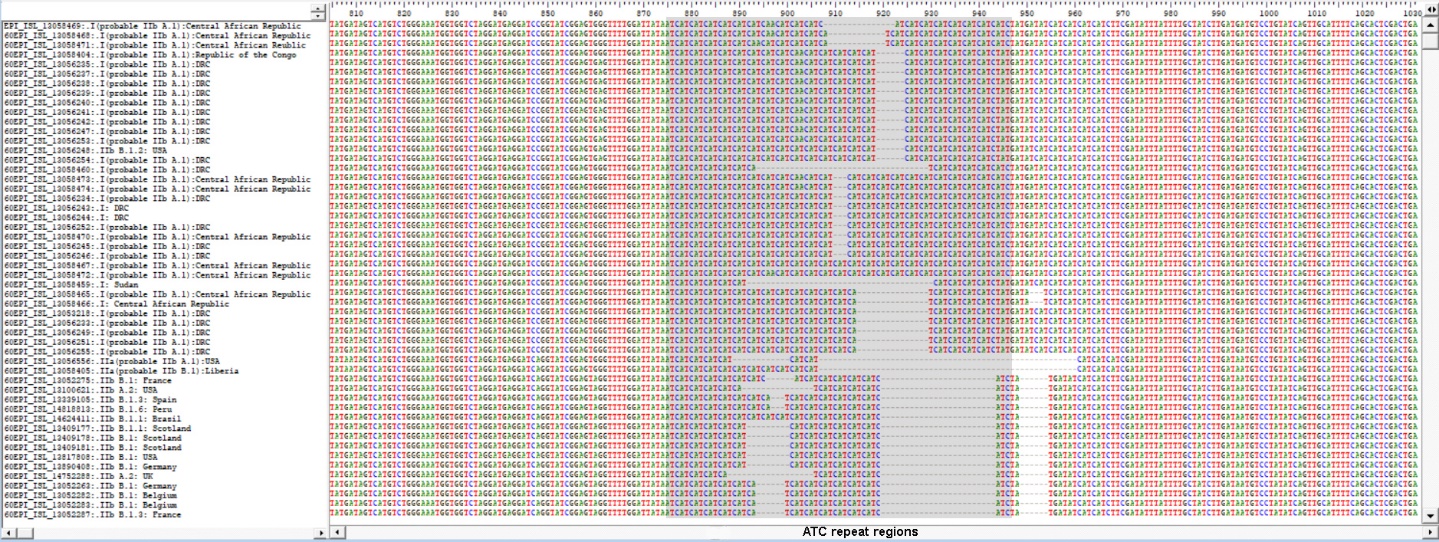


A supplementary figure is provided, displaying the multiple sequence alignment of the top 50 homologous areas of gene *OPG153* of MPXV. The image highlights the contraction of the ATC motif in recent lineages compared to ancestral ones. The shaded alignment sections depict the area that includes the varied ATC motifs, specifically from position 875 to 987.
